# Spatial variations and influencing factors of Cumulative Health Deficit Index of elderly in China

**DOI:** 10.1186/s41043-023-00403-4

**Published:** 2023-07-11

**Authors:** Liuchun Xiang, Masaaki Yamada, Wenmeng Feng, Dan Li, Haisong Nie

**Affiliations:** 1 United Graduate School of Agricultural Science, Tokyo University of Agriculture and Technology, Tokyo, 1838509 Japan; 2 Development Research Center of the State Council, Beijing, 100010 China; 3 Division of International Environmental and Agricultural Science, Institute of Agriculture, Tokyo University of Agriculture and Technology, Tokyo, 1838509 Japan

**Keywords:** Elderly, Cumulative Health Deficit Index, CHDI, Geodetector, Spatial variation

## Abstract

**Background:**

With the acceleration of aging and urbanization, how to make cities more age-friendly has become a hot topic. During the long-term demographic transition, the health of the elderly has become an important consideration in urban planning and management. The health of the elderly is a complex issue. However, previous studies have mainly focused on the health defects related to disease incidence, loss of function, mortality, etc., yet a comprehensive evaluation of health status is lacking. The Cumulative Health Deficit Index (CHDI) is a composite index that combines psychological and physiological indicators. Health deficits can reduce the quality of life of the elderly and increase the burden on families, cities and even society, so it is indispensable to understand the individual factors and regional factors that affect CHDI. The research on the spatial differentiation of CHDI and its driving factors can provide scientific geographic information basis for the construction of age-friendly cities and healthy city planning. It also has great significance in narrowing the differences in the health status among regions and reducing the overall burden of the country.

**Methods:**

This research analyzed a nationwide dataset, the China Longitudinal Aging Social Survey in 2018 conducted by the Renmin University of China, which contained 11,418 elderly aged 60 years and older from 28 provinces/municipalities/autonomous regions that represent 95% of the population in mainland China. The Cumulative Health Deficit Index (CHDI) was the first time constructed using the entropy-TOPSIS method to evaluate the health status of the elderly. Entropy-TOPSIS is to quantify the importance of each indicator by calculating the entropy value to improve the reliability and accuracy of the results and avoid the influence of previous researchers’ subjective assignments and model assumptions on the results. The selected variables include physical health 27 indicators (self-rated health, basic mobility assessment, daily activity ability, disease and treatment) and mental health 36 indicators (cognitive ability, depression and loneliness, social adjustment, and filial piety concept). The research used the Geodetector methods (factor detection and interaction detection) that combined individual and regional indicators to analyze the spatial variation characters and reveal the driving factors of CHDI.

**Results:**

The weight of mental health indicators (75.73) is three times that of physical health indicators (24.27), and its composition formula is: CHDI value = (14.77% disease and treatment + 5.54% daily activity ability + 2.14% health self-assessment + 1.81% basic mobility assessment) + (33.37% depression and loneliness + 25.21% cognitive ability + 12.46% social adjustment + 4.7% filial piety). Individual CHDI was more associated with age and was more evident in females than males. CHDI average values show the distribution trend of Hu Line (HL) in the geographic information graph that the CHDI in West HL regions are lower than in the East HL regions. The highest CHDI cities are in Shanxi, Jiangsu, and Hubei, whereas the lowest CHDI cities are Inner Mongolia, Hunan and Anhui. The geographical distribution maps of the 5-levels of CHDI levels show very different CHDI classification levels among the elderly in the same region. Further, the top three influential factors are personal income, empty nest, aged 80+, and regional factors also obviously affect CHDI values, such as the proportion participating in insurance, population density, and GDP. The two different factors in individual and regional factors all show a two-factor interaction effect, and enhancement or nonlinear enhancement. The top three ranks are personal income ∩ quality of air (0.94), personal income ∩ GDP (0.94), and personal income ∩ urbanization rate (0.87).

**Conclusions:**

CHDI is a subjective and objective comprehensive index, and mental indicators are primary factors. Thus attaching importance to the psychological care of the elderly is the key to building a healthy aging society. The large individual heterogeneity and spatial differentiation of CHDI in the elderly were demonstrated by map visualization. The analysis of the influencing factors of CHDI by the Geodetector method proves that spatial differentiation is mainly affected by individual economic and social security factors, but also by the interaction with regional factors such as quality of air, GDP, and urbanization rate. This research fills a gap in the elderly health status in the field of spatial geography. The results can provide empirical evidence for policymakers to take measures according to local conditions to improve the health status of the elderly according to regional differences in physical and mental conditions. It also plays a guiding role for the country in balancing regional economic development, promoting healthy and sustainable urban development, and creating age-friendly cities.

## Background

With the acceleration of aging and urbanization, it has become a hot topic for scholars to make communities or cities more age-friendly. The WHO’s work on age-friendly cities has identified key factors affecting the health of elderly. The Outline of the Healthy China 2030 Plan in 2016 mentioned that healthy city is an important part of the plan, with a view to providing better health services to the elderly. Healthy and age-friendly cities are an integral part of urban development and contribute to the sustainable development of cities. In the context of long-term aging and demographic transition, the health of elderly is a crucial consideration in urban planning and management. Different from other countries, China has the characteristics of unbalanced regional economic development, diverse geographical environment and wide distribution of the elderly population. Therefore, it is essential to analyze the geographical distribution of the health conditions of the elderly and analyze the spatial differentiation characteristics and driving factors of the health status of the elderly in combination with individual factors and regional factors.

The WHO [3] defines health to mean physical health, mental health, and social adaptability. Measuring the health status of the elderly plays a crucial role in achieving healthy aging. The indicators of elderly health status commonly used in the world are Activities of Daily Life (ADL), Instrumental-based Activities of Daily Life (IADL), Mini-mental state examination (MMSE scale), Frailty Index (FI) and Cumulative Health Deficit Index (CHDI) to measure physical health, mental health, frailty conditions and overall health status, respectively. FI is an evolving concept that can be divided into two functions, phenotypes and cumulative frailty index [4, 5, 14, 15]. The comprehensive health index of CHDI is derived from FI by combining both subjective and objective indicators. CDHI is widely used in the study of healthy aging. It aggregates the impairment scores of different dimensions of health variables, including cognitive function, ADL, IADL, physical activity ability, self-rated health, psychological stress, serious illness and various chronic diseases. Mitnitski and Mitnitski et al. [12, 13] and based on the research of the USA, Canada and other countries showed that the estimated CHDI was very consistent as long as the variables used to construct the CHDI among different populations reach a certain amount [1]. Gu also used Chinese national elderly population data to compare CHDI with that of other countries, revealing the validity and reliability of this index [7]. Many studies have shown that the CHDI has good predictive power in reflecting health status, health service use, public health management and so on [8, 9, 11]. In this study, 63 variables of CHDI with multiple dimensions (mental dimension and physical dimension) were selected based on national data based on prior literature and social-cultural background, which enriched the diversity of indicators and supplemented the comprehensive indicators in the field of empirical research on CHDI.

Th entropy-TOPSIS method was the first time used to construct CHDI in this research. The method quantifies the importance of each indicator by calculating the information entropy of each indicator, which is innovative and scientific in the construction of indicators of the health status of the elderly. This study visualized of the spatial distribution of the average CHDI and 5-level CHDI through geographic information system that visually evaluated the variations in the average health level among regions and the discrepancy in the graded health level within regions. It fills the blank in the spatial geographic research on the health status of the elderly.

Geodetector is a new statistical method to detect spatial variation and reveal the driving factors [17]. The advantages of Geodetector are: (1) it can detect numerical data or qualitative data; (2) it can not only detect whether the two factors interact with the dependent variable but also can analyze the strength, direction, linearity or nonlinearity of the interaction. As long as the two factors have a relationship, it can be tested. This method has been widely used in the research of diseases, land use, ecology, environment, regional economic planning and so on. This study is the first time to research the spatial differentiation characteristics and driving factors of the health conditions of the elderly based on the Geodetector method. It can comprehensively grasp the independent role or interaction of individual factors and regional factors affecting the health status.

## Methods

### Data source

The China Longitudinal Aging Social Survey (CLASS) is a large-scale national continuous social survey project that has been conducted by the China Survey and Data Center of Renmin University of China since 2014 and followed up every two years. The survey items include health status, care needs, economic conditions, social insurance, employment, family pension resources, community pension facilities, community service, pension planning, pension mode choice, and attitude of elderly toward aging. The survey uses stratified multistage probability sampling, which has universality and authority. The data from 28 provinces/municipalities/autonomous regions that represent 95% of Chinese mainland population, aged 60 years and older and in total 11,418 samples (http://class.ruc.edu.cn/xmjz/cysj.htm). The research selected CLASS data in 2018.

### Research methods

**Fig. 1:**
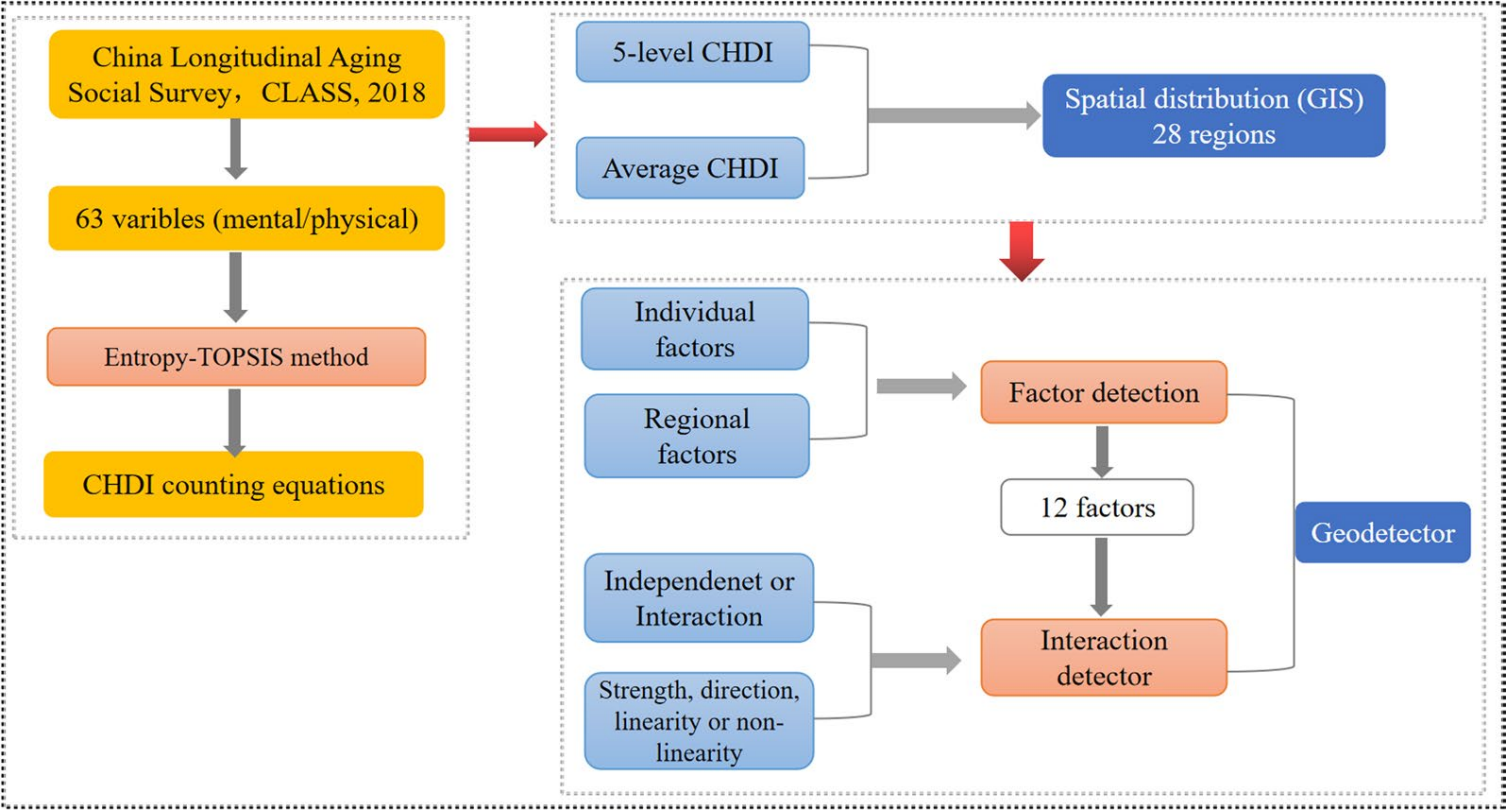
Research process

The CHDI was constructed by entropy-TOPSIS method and then counting the average CHDI and 5-levles CHDI in 28 regions visualized by ArcGIS. Combining individual variables and regional variables were analyzed by Geodetector (factor detection and interaction detection). The research process is below in Fig. 1.

#### Entropy-TOPSIS method construct CHDI

In previous studies, when calculating the health cumulative deficit index, each variable was coded first, health 0 and health 1. The 40 deficit index, its theoretical deficit score is 40 points. Assuming that an individual’s total deficit score is 10 points, the personal health cumulative deficit index = 10 points/theoretical deficit full score of 40 = 0.25. The exponential minimum value was 0, and the maximum value was 1. An index of 0 indicated that the elderly person was healthy on all measured health indices. A score of 1 indicated that the elderly person was considered unhealthy on all variables [7]. This calculation method presumes that each index has an equal impact on health, but in actuality, each index varies from individual to individual.

The entropy-TOPSIS method was used to establish the CHDI model to distinguish the weight of each influential factors. The TOPSIS method compares the relative distance of each measured object with the maximum value and the minimum value, which has the advantages of simple calculation and reasonable results [22, 25]. The entropy-TOPSIS method combines the advantages of the entropy weight method and the TOPSIS method for more objective and reasonable measurement results. The detailed process is as follows: First, weight each measured index by the entropy weight method, and then quantify the index by using the TOPSIS method. The index weight value is based on the information reflected by the variation degree of the data of each measure index. The entropy value determines the dispersion degree of the index. The smaller the entropy value, the greater the difference degree of the index, that is, the greater the role of the index in the evaluation process, the greater the weight of the index. The entropy weight in this research was calculated by the degree of difference of each index, and more objective results were obtained by weighting the entropy weight and the gray correlation coefficient [2].

Some of the indicators selected are positively correlated with CHDI value, while others are negatively correlated with CHDI value. To eliminate the effect of different symbols and dimensions on the calculation, first use the linear function to process the observed index value. The first index and the first evaluation object (= 1,2,…,; = 1,2,…,…) are set to be scored as follows. Above all, the positive indicators are scored by using the following formula:

1 $$q_{ij} = \frac{{p_{ij} - \mathop {\min }\limits_{{\left( {1 \ll j \ll n} \right)}} \left( {p_{ij} } \right)}}{{\mathop {\max }\limits_{{\left( {1 \ll j \ll n} \right)}} \left( {p_{ij} } \right) - \mathop {\min \left( {p_{ij} } \right)}\limits_{1 \ll j \ll n} }}\quad (i = 1,2, \ldots ,m)$$

The negative indicators are scored by

2 $$\frac{{\mathop {\max }\limits_{{\left( {1 \ll j \ll n} \right)}} \left( {p_{ij} } \right) - p_{ij} }}{{\mathop {\max }\limits_{{\left( {1 \ll j \ll n} \right)}} \left( {p_{ij} } \right) - \mathop {\min \left( {p_{ij} } \right)}\limits_{1 \le j \le n} }}\quad \left( {i = 1,2, \ldots ,m} \right)$$

The intermediate indicators are scored by

3 $$M = \max \left\{ {\left| {q_{i} - q_{{{\text{best}}}} } \right|} \right\};\quad \tilde{q}_{i} = 1 - \frac{{\left| {q_{i} - q_{{{\text{best}}}} } \right|}}{M}$$

Secondly, the entropy value of each indicator is calculated. Set the proportion of the first evaluation index as the entropy value of the first evaluation index, there is:

4 $$f_{ij} = \frac{{p_{ij} }}{{\sum\nolimits_{n = 1}^{n} {p_{ij} } }}$$

5 $$e_{i} = \frac{1}{\ln n}\sum\nolimits_{n = 1}^{n} {\ln f_{ij} }$$

Let *w*_*i*_ be the entropy weight of the *i* indicator, then:

6 $$w_{i} = \frac{{1 - e_{i} }}{{\sum\nolimits_{i = 1}^{m} {\left( {1 - e_{i} } \right)} }}$$

Finally, the total score of the *j* evaluation object is calculated:

7 $$s_{i} = \sum\nolimits_{i = 1}^{m} {w_{i} q_{ij} } \times 100$$

The CHDI values of individual are calculated. Firstly, use formulas (4) and (5) to obtain the entropy value of each 3-Level index. Next, calculate the sum of the entropy weights of the 3-Level. Finally, formulas (1), (2), (3), and (6), (7) are used to calculate the sum of the product of each normalized score and each index of the 2-Level index. The entropy weight of 1-Level 1 and 2-Level indicators are shown in Table 3 (the entropy weight of 3-Level indicators are in Appendix Table).

This research counted the average CHDI and 5-level CHDI in 28 regions in China and visualized in the maps. JENKS in ArcGIS is used to convert spatial surface data into software input data. For irregular surface data (such as administrative areas), the sample points of spatial system distribution (such as fixed spacing regular lattice) are generated by the lattice method, and the information of the independent variable and dependent variable of the location of each lattice is extracted. Finally, the extracted data are run in the software as input data [24].

#### Geodetector

The advantage of Geodetector is that there are no excessive assumptions. Traditional statistical analysis methods deal with the limitations of category variables, which are risk detection, factor detection, ecological detection, and interactive detection. Risk detection mainly explores where the risk area is located, factor detection is used to identify what factors cause the risk, ecological detection indicates the relative importance of risk factors, and interactive detection is used to determine whether the influencing factors work independently or together [18, 23]. This research was analyzed by factor detection and interactive detection.

This research summarized variables from previous literature to screen for factors in the individual and regional dimensions. The two dimensions’ factors include a total of 23. Individual indicators include age, gender, spouse, hukou, aged 80+, level of education, proportion of only child, empty nest rate, personal income, household income, proportion participating in insurance, and employment rate. Regional indicators include GDP, unemployment, urbanization rate, population density, literacy, dependency rate, annual average temperature, air quality, annual precipitation, health institutions, and number of beds per thousand people. It used the exploratory factor analysis method with characteristic values greater than 1, and the maximum variance method was selected for factor rotation. Then, 12 principal component factors were extracted (general standard with KMO value of 0.756 and Bartlett significance coefficient of 0.00, indicating that the variable is suitable for factor analysis). The cumulative variance contribution rate reached 79.8, which can better explain most of the information on the variables [19].

(1) Differentiation and factor detection: Detect the spatial differentiation of CHDI and to what extent each factor *X* explains the spatial differentiation of attribute CHDI. *q* value is used to measure, expressed as:

8 $$q = 1 - \frac{{\sum\nolimits_{h = 1}^{L} {N_{{h\sigma^{2} h}} } }}{{N\sigma^{2} }}\quad \left( {h = 1, \ldots } \right)$$

The range of *q* is [0, 1]. The larger the value of *q* is, the more distinct the spatial differentiation of CHDI, and the stronger the explanatory power of independent variable *X* is for CHDI, the weaker the otherwise. In extreme cases, a *q* value of 1 indicates that factor *X* completely controls the spatial distribution of CHDI, a *q* value of 0 indicates that factor *X* has no relationship with CHDI, and a *q* value indicates that factor *X* explains 100 × *q*% CHDI. A simple transformation of q values satisfies a non-central F-distribution:

9 $$F = \frac{N - L}{{L - 1}}\frac{q}{1 - q} \sim F\left( {L - 1,\;N - L;\;\lambda } \right)$$

10 $$\lambda = \frac{1}{{\sigma^{2} }}\left[ {\sum\nolimits_{h = 1}^{L} {\overline{Y}_{h}^{2} } - \frac{1}{N}\left( {\sum\nolimits_{h = 1}^{L} {\sqrt {N_{h} \overline{Y}_{h} } } } \right)^{2} } \right]$$

where *λ* is a non-central parameter and *Y*_*h*_ is the mean of layer *h*。

(2) *Interaction detection* Identify the interaction between different risk factors *X*_s_; i.e., assess whether the combined action of factors *X*_1_ and *X*_2_ will increase or decrease the explanatory power of CHDI, or whether these factors have independent effects on CHDI. The evaluation method is to first calculate the q values of two factors *X*_1_ and* X*_2_* f*or CHDI: *q *(*X*_*1*_) and *q*(*X*_*2*_), respectively, calculate the q values of their interaction: *q*(*X*_*1*_* ∩ X*_*2*_), and compare *q*(*X*_*1*_), *q*(*X*_*2*_) with *q*(*X*_*1*_* ∩ X*_*2*_). The relationship between the two factors can be divided into the following categories (Table 1).

**Table 1:** Basis for judging two-factor interaction patterns

Type	Basis of judgment	Interaction
1	$q\left( { \, X_{1} \cap X_{2} } \right) < Min\left( { \, q\left( { \, X_{1} } \right),\;q\left( { \, X_{2} } \right)} \right)$	Nonlinear weakening
2	${\text{Min}}\left( { \, q\left( { \, X_{1} } \right),\;q\left( { \, X_{2} } \right)} \right) < q\left( { \, X_{1} \cap X_{2} } \right){\text{ < Max}}\left( {q\left( { \, X_{1} } \right),\;q\left( {X_{2} } \right)} \right)$	Single nonlinear enhancement
3	$q\left( { \, X_{1} \cap X_{2} } \right) < {\text{Max}}\left( {q\left( { \, X_{1} } \right),\;q\left( { \, X_{2} } \right)} \right)$	Double enhancement
4	$q\left( { \, X_{1} \cap X_{2} } \right) = q\left( { \, X_{1} } \right) + q\left( { \, X_{2} } \right)$	Independence
5	$q\left( { \, X_{1} \cap X_{2} } \right) > q\left( { \, X_{1} } \right) + q\left( { \, X_{2} } \right)$	Nonlinear enhancement

## 3. Results

### 3.1 Descriptive Statistics

The mean age was 71.45 years and the ratio of males and females was 50.2% and 49.9%, respectively (Table 2). The rural population accounted for 42.3%. The proportion of having a spouse was 69.3%. The overall sample had a low education level, with 26.5% being illiterate and 67.2% being below primary school. 9389 valid data show that the average personal annual personal income is 749.91 RMB.

**Table 2:** Descriptive statistics

Variable	Describe	Mean/Percentage
Age	Average age of total sample	71.45
Sex	Proportion of the man sample	50.2%
Hukou	Proportion of the rural household registration sample	42.3%
Spouse	Proportion of having a spouse sample	69.3%
Level of education	Proportion of unattended (illiterate) samples	26.5%
Personal income	Median annual income of the individuals (RMB/year)	5500

### Results of CDHI values

The total weight of mental health accounts for 75.73% of CHDI, and highest weights are depression and loneliness (33.37%), followed by cognitive ability (25.21%), social adjustment (12.46%), and filial piety (4.7%) (Appendix Table). The total weight of physical health is only 24.47%, in which the indicator of disease and treatment has the largest weight (14.77%), followed by daily activity ability assessment (5.54%). The weights of the indicators of health self-assessment and basic mobility assessment are relatively small (2.14% and 1.82%, respectively). Then, the counting equations for the CHDI values as below:

- CHDI value = 24.27% physical health + 75.73% mental health;
- CHDI value = (14.77% disease and treatment + 5.54% daily activity ability + 2.14% health self-assessment + 1.81% basic mobility assessment) + (33.37% depression and loneliness + 25.21% cognitive ability + 12.46% social adjustment + 4.7% filial piety).

**Fig. 2:**
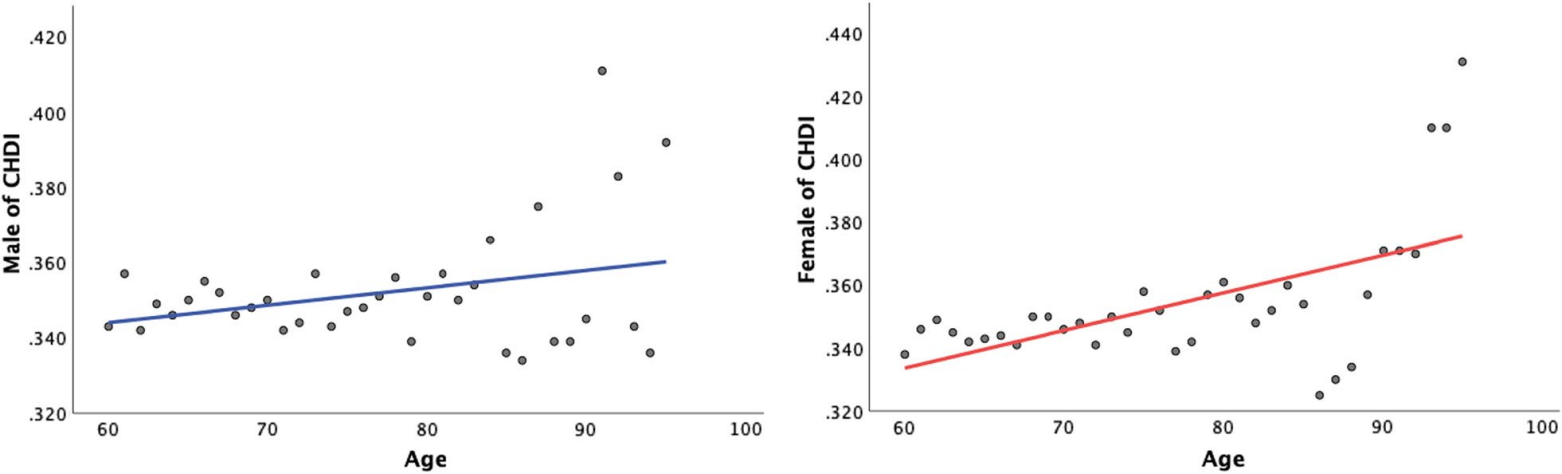
Age analysis of CHDI by gender

The average CHDI value of Chinese elderly was 33.82, the minimum value was 10.28, and the maximum value was 64.97, showing a significant difference in health status. As shown in the analysis by gender (Fig. 2), women consistently had higher health deficits than men. The CHDI values of women and men increase linearly with age. Men had a lower correlation with age (*P* = 0.352), and women had a higher correlation with age (*P* = 0.001).

### Spatial distribution pattern and characteristic analysis

#### Average CHDI

In order to explore the inter-provincial distribution and differences in CHDI value among the elderly in China, average value of each region is measured. The ranking of the resulting levels is shown in Table 3. The five regions with the lowest scores were Inner Mongolia (28.91), Hunan (29.06), Anhui (29.82), Sichuan (30.95), and Guangdong (31.23), which indicated that the health status was relatively good. The five provinces (or regions) with the highest scores were Heilongjiang (37.49), Fujian (38.20), Hubei (39.07), Shaanxi (40.30), and Jiangsu (42.54), indicating relatively poor health conditions.

**Table 3:** Average CHDI value of 28 regions

Province/Province-level region/Province-level division	CHDI		Province/region	CHDI	
	Average value	Rank		Average value	Rank
Inner Mongolia	28.91	1	Ningxia	35.81	15
Hunan	29.06	2	Zhejiang	35.85	16
Anhui	29.82	3	Jiangxi	35.95	17
Sichuan	30.95	4	Hebei	36.15	18
Guangdong	31.23	5	Yunnan	36.49	19
Beijing	31.48	6	Shandong	36.60	20
Tianjin	32.12	7	Chongqing	36.80	21
Liaoning	32.21	8	Guangxi	36.91	22
Gansu	32.86	9	Shanxi	36.93	23
Jilin	33.14	10	Heilongjiang	37.49	24
Henan	33.54	11	Fujian	38.20	25
Qinghai	33.63	12	Hubei	39.07	26
Shanghai	33.94	13	Shaanxi	40.30	27
Guizhou	35.12	14	Jiangsu	42.54	28

**Fig. 3:**
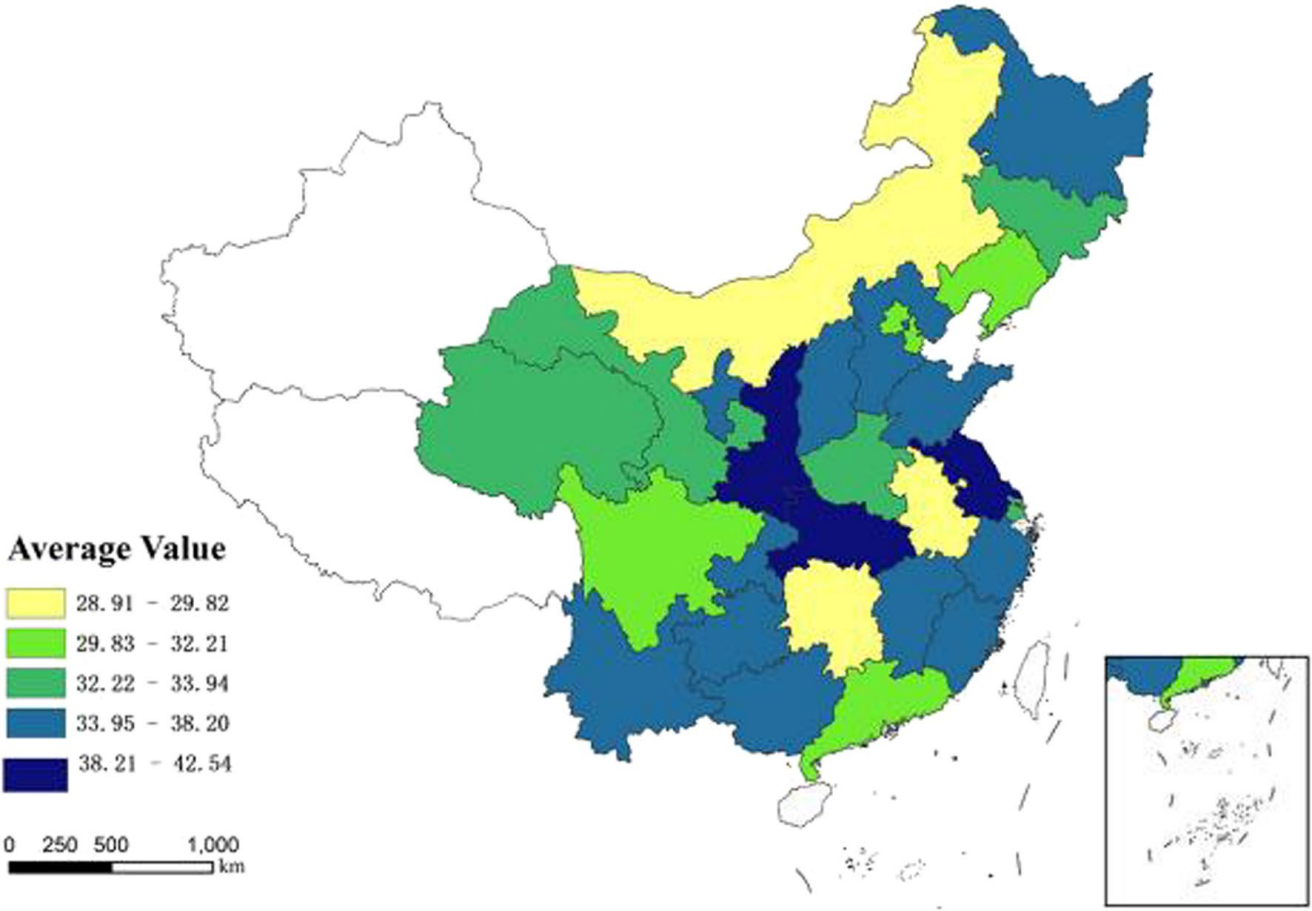
The average CHDI

The results verified that the CHDI value is different in each region of China. The distribution trend of the Hu Line (HL) is obvious [10, 21]. HL also known as Chinese Hu HuanYong Line. It is a well-known natural geographical dividing line. It bisects China into two regions: the east of Hu line (East HL) and the west of Hu line (West HL). East HL is 36% of the territory and is occupied by 96% of the population. West HL is 64% and 4%, respectively. The HL reveals the spatial distribution pattern of population density and the dividing line for both environment and human activities in China was unchanged since the 1230 s. Most of the regions with the highest CHDI are located in the East HL, and most of the regions with the lowest CHDI are distributed in the West HL (Fig. 3). It means that the elderly health status in West HL regions are better than East HL regions.

#### The 5-level CHDI

The CHDI results are significantly different (10.28–64.97) and the average CHDI can well show the variation among regions. However, the differentiation of same region needs to be reflected by five levels of classification. Therefore, quantitative stratification to divide CHDI value into five levels: the lowest CHDI (10.28–28.11), lower CHDI (28.12–32.17), middle CHDI (32.18–36.27), higher CHDI (36.27–41.51), and the highest CHDI (41.5–64.97). Then calculate the regional distribution of the number of people at each level (Table 4).

**Table 4:** CHDI Value classification standard

CHDI Level	Level 1	Level 2	Level 3	Level 4	Level 5
	Lowest deficit	Lower deficit	Middle deficit	Higher deficit	Highest deficit
CHDI Value	10.28 ≤ 28.11	28.12–32.17	32.1–36.27	36.27–41.51	41.5–64.97

**Fig. 4:**
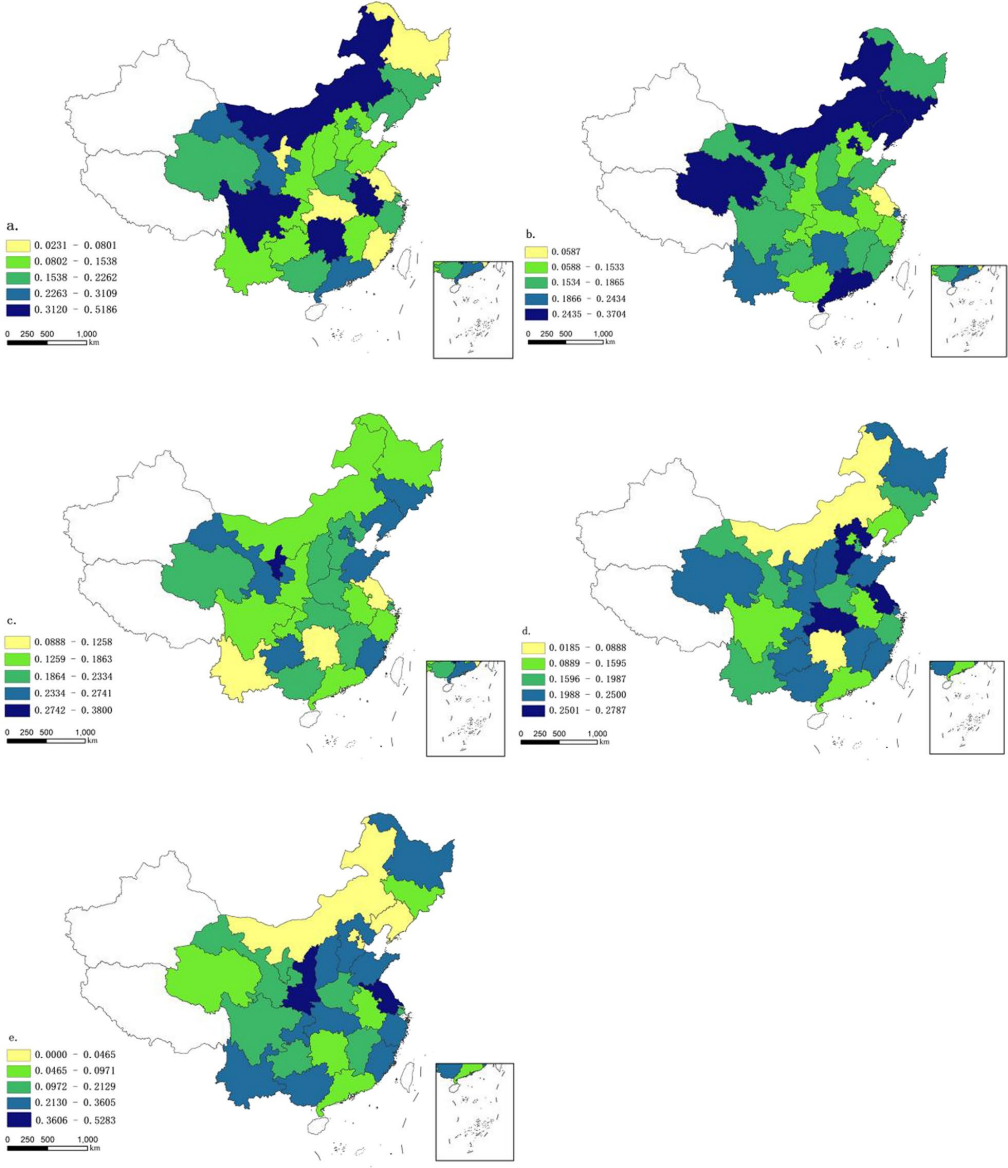
The 5-level CHDI (a: Level 1; b: Level 2; c: Level 3; d: Level 4; e: Level 5)

Figure 4a–e shows the proportional distribution of different levels of population in the sample population of each region. The darker the color, the more represented by the number of people there are. Figure 4a shows the spatial distribution of the proportion of level 1 people among the regions, (Population of Level 1/population of sample), with the highest proportion being Inner Mongolia, Sichuan, Hunan, and Anhui; Fig. 4b shows the level 2 with the highest proportion being Inner Mongolia, Jilin, Liaoning, Beijing, Tianjin, Qinghai, Guangdong; Fig. 4c shows the level 3 with the highest proportion being Ningxia; Fig. 4d shows the level 4 with the highest proportion being Hebei, Jiangsu, Hubei; Fig. 4e shows the level 5 with the highest proportion being Shaanxi, Jiangsu.

### Geodetector analysis results

#### Factor detection

The partition of space is the result of the integrated action of multifactors. The factor detector results indicate that the larger the *q*-value of the factor, the greater the explanatory force of the factor for spatial separation. From the data results, the individual factors that affect CHDI with largest spatial differentiation are personal income, empty nest and aged 80+, while the regional factors are proportion participating insurance, density population and GDP (Table 5).

**Table 5:** Factor detection results of spatial differentiation of CHDI value

Factor	Abbreviations	*q* statistic	Rank	Descriptions
Personal income	II	0.54	1	Average annual personal income (RMB)
Empty nest	EN	0.52	2	The ratio of people living alone (%)
Aged 80+	Aged	0.34	3	The ratio of elderly aged 80+
Household Income	FI	0.31	4	Average monthly household income (RMB)
Sex	SEX	0.24	5	Proportion of the man population
Person Living Together	PLT	0.24	6	Average number of person living together
Proportion participating insurance	INS	0.24	7	Region workers’ insurance participation rate is (%)
Density of Population	DP	0.18	8	Population divided by area
GDP	GDP	0.15	9	Province GDP (Dollars)
Quality of Air	QA	0.15	10	The days with better air quality or above level 2 in cities accounted for in 2018 (%)
Proportion of only child	NC	0.12	11	The ratio of the only child is (%)
Urbanization rate	UR	0.07	12	The urban population is divided by the total population (%)

#### Interaction detector

Interaction detection is performed by comparing the degree to which a factor variable is greater or smaller than the individual factor. There are five types of two-factor X1 and X2 interactions: reduced nonlinearity, two-factor enhancement, reduced single-factor nonlinearity, enhanced nonlinearity, and mutual independence. The results of the interaction detection analysis with these 12 influencing factors showed that the interaction between any two factors was two-factor enhancement or nonlinear enhancement and there was no independent or weakened relationship. This means that the interaction of any two factors affects CHDI value more than a single factor. The higher the interaction *q* value, the greater the interaction between the two corresponding factors that affect CHDI [20].

**Fig. 5:**
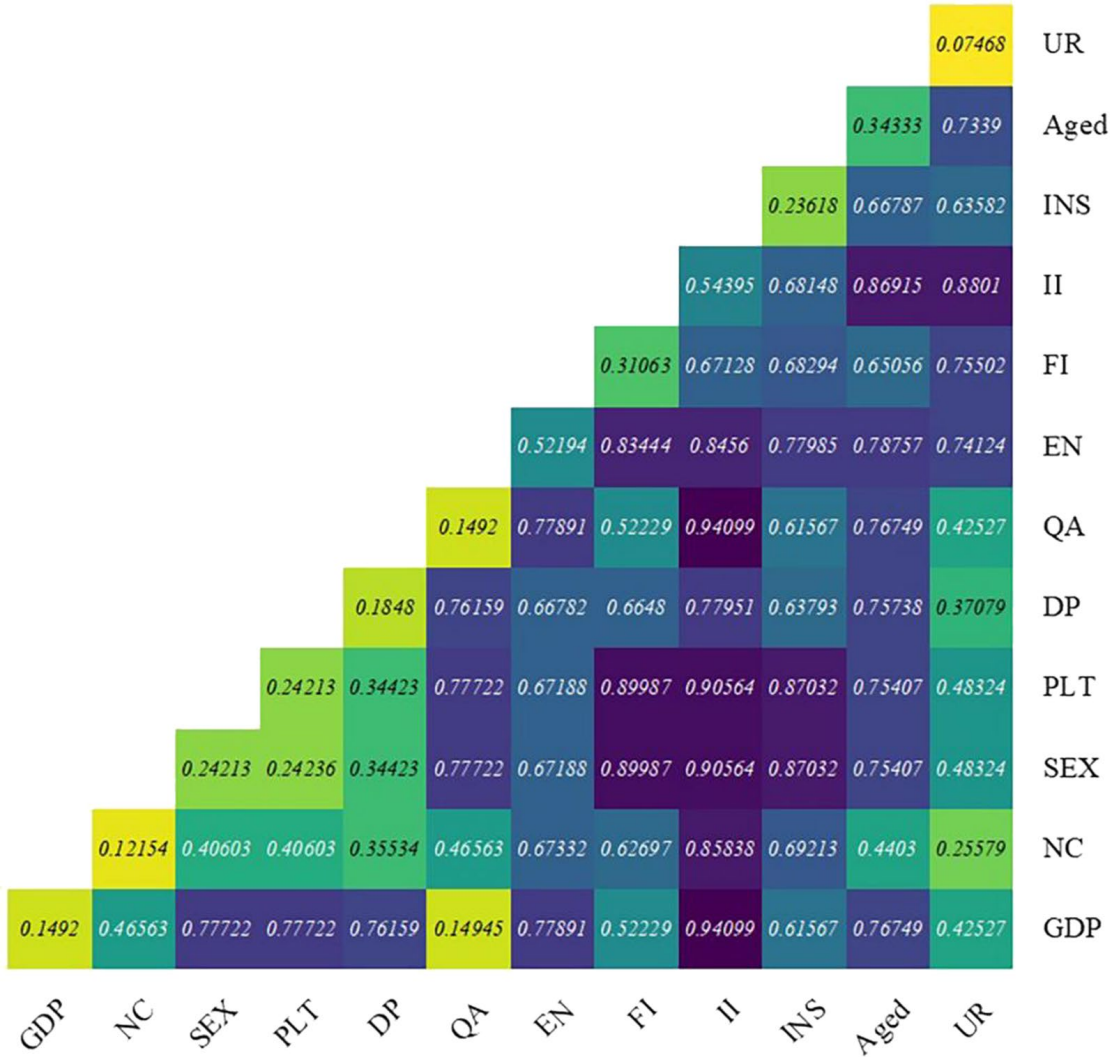
Interaction detector analysis result

The interaction detector results of individual and regional two dimensions’ factors show that two-factor interaction effect and enhancement or nonlinear enhancement. The strongest effect of the interaction on CHDI was personal income ∩ Quality of air (0.94), personal income ∩ GDP (0.94), personal income ∩ Urbanization Rate (0.87) (Fig. 5). Personal income had the highest *q* value among the interaction effects and all other factors, which was associated with the higher uni-factorial explanatory force of personal income on CHDI. Second, personal income has the strongest combined effect with quality of air, GDP and urbanization rate.

## Discussion

The core factor affecting CHDI values is the psychological aspect, which was three times more likely to have an effect than the physical aspect. Among them, depression, loneliness, and cognitive ability accounted for the largest proportion. Diseases and coping methods and social adaptation accounted for one-third of the health of the elderly. However, self-rated health, ADL, and IADL, indicators commonly used by researchers, accounted for less than 10%, which had little impact on the health of the elderly. The traditional concept of filial piety only accounted for 4.7% of the impact on CHDI.

The results showed significant differences of CHDI in Chinese elderly indicating high individual heterogeneity. The CHDI increase linearly with age and health deficits of women are consistently higher than men, which was consistent with previous research. Mitnitski’s study based on data from four developed countries in the USA, Canada, Australia, and Sweden, showed that women were on average with more health deficit than men at all ages [13]. The slope was close between both men and women, and the individual correlation with age was very high (*r* = 0.95, *P* < 001). Gu’s study [6, 7] used the responses to the 2002 Chinese Longevity and Health Survey, and the results were very close to that of Mitnitski’s, although the two studies used different variables.

The estimated CHDI values were visualized by ArcGIS, which indicated that the average value of CHDI appeared in the HL distribution tendency, and CHDI average values in the West HL were smaller than that in the East HL. The provinces with the highest average deficit are Jiang su, Shanxi and Hubei; those with the lowest are Inner Mongolia, Hunan, and Anhui. The average value of CHDI may be related to population economy, geographical environment and social-culture which can be afford proof for further studied. After dividing the CHDI values into five levels, the difference in health between the provinces became more pronounced. This also demonstrates the spatial heterogeneity in the health status of the elderly. The same patterns were observed in Europe; there were distinct differences in elderly health in northern, southern, and western Europe, and the main influencing factors differed depending on the region [16].

The factor detection results showed that both individual and regional factors affected the ranking of the *q* values. In addition to individual indicators such as personal income, empty nest and aged 80+, the results also revealed the impact of regional factors on elderly health conditions in spatial variation. However, the explanatory power of individual factors is higher than that of regional factors. The interaction detector analysis results showed that the two dimension factors’ interaction were significant, particularly personal income ∩ quality of air (0.94), personal income ∩ GDP (0.94) and personal income ∩ urbanization rate (0.87). Although regional factors have a weaker influence on the elderly health than individual factors, interaction was evident.

Nearly half of the respondents in the research were in the empty nest, and the decrease in family support poses a great threat to the health of the elderly. The results indicated that family factors were a significant influence on the elderly. In the factor detection, empty nest, household income, and the number of cohabitants are all ranked relatively high. Among the interaction factors are Personal income ∩ Person Living Together (0.91), household income ∩ Sex (0.90), household income ∩ Person Living Together (0.90), and Insurance ∩ Person Living Together (0.87). Thus, the health of the elderly is closely related to family factors.

## Conclusions

The elderly health is a complex and comprehensive social problem. CHDI is a subjective and objective comprehensive index and mental indicators are primary factors. Thus attaching importance to psychological care of the elderly is the key to building a healthy aging society. The health status may also be affected by the different geographical environments, social culture and subjective feelings. The large individual heterogeneity and spatial differentiation of CHDI were demonstrated by map visualization. The analysis of the influencing factors of CHDI by Geodetector method proves that the spatial differentiation is mainly affected by individual factors but also by the interaction with regional factors such as quality of air, GDP, and urbanization rate. This research fills a gap of the elderly health status in the field of spatial geography. The results can provide empirical evidence for policymakers to take measures according to local environment and cultural background to improve the health status of the elderly according to regional differences in physical and mental conditions. It also plays a guiding role for the country in balancing regional economic development, promoting healthy and sustainable urban development, and creating age-friendly cities.

## Data Availability

The CLASS data supporting this study's findings are available from the Population Development Studies Center of the Renmin University of China.

## Appendix

**Table:** Entropy weight of each indicator in the questionnaire of CLASS 2018

Health	Indicators	Question number	Question	Entropy value	Entropy weight	Entropy weight	Entropy weight
1-Level	2-Level	3-Level			1-Level	2-Level	3-Level
Physical Health	Self-rated Health	B1	How do you feel about your current physical health?	0.990209	0.2427248	0.021371	0.009636
		B2	What do you think of your health status, compared to your peers?	0.991262			0.008599
		B3	How has your health status changed compared to last year?	0.997660			0.002303
		BMI(Average Value)	BMI = weight / height ^2^	0.999153			0.000833
	Basic Mobility Assessment(containing ADL 6 indicators)	B4-1	Can you use your phone for yourself?	0.994341		0.018193	0.005569
		B4-2	Can you tidy yourself up (combing your hair, shaving, making makeup, etc.)?	0.998213			0.001758
		B4-3	Can you dress by yourself?	0.998719			0.001261
		B4-4	Can you bathe yourself (shower or bath)?	0.997799			0.002166
		B4-5	Can you eat by yourself?	0.999042			0.000943
		B4-6	Can you take the medicine yourself?	0.998731			0.001249
		B4-7	Do you have urinary incontinence?	0.998380			0.001594
		B4-8	Do you have fecal incontinence?	0.999184			0.000803
		B4-9	Can you go to the toilet by yourself?	0.998895			0.001087
		B4-10	Can you move from the bed to the bedside chair yourself?	0.999028			0.000957
		B4-11	Can you walk indoors?	0.999181			0.000806
	Daily Activity Ability Assessment (containing IADL 10 indicators)	B6-1	Can you get up and down the stairs (steps)?	0.997096		0.055417	0.002857
		B6-2	Have you ever fallen down in the past 12 months?	0.996521			0.003423
		B6-3	Have you ever fallen down in the past 12 months?	0.998143			0.001827
		B6-4	Can you take public transportation (e.g. bus, subway) by yourself?	0.995243			0.004682
		B6-5	Can you go shopping by yourself?	0.995491			0.004437
		B6-6	Can you manage your own money?	0.991741			0.008128
		B6-7	Can you lift something weighing 10 kg (5 kg)?	0.978065			0.021586
		B6-8	Can you cook the food for yourself?	0.995634			0.004296
		B6-9	Can you do the housework by yourself?	0.995752			0.004181
	Disease and response ( chronic disease, illness treatment, hospitalization)	B9	What chronic diseases do you have?	0.859159		0.147744	0.138601
		B11	How do you usually deal with your minor illness?	0.990881			0.008974
		B12-1	How many times have you been hospitalized in the last two years?	0.999829			0.000168
Mental Health	Cognitive ability	E1-1–1	What day is it today? (either lunar calendar or solar calendar)	0.993656	0.757275126	0.252051	0.006243
		E1-1–2	What is the name of this community / village?(Neighborhood committee or community / village name)	0.997739			0.002225
		E1-1–3	When is National Day? (October 1)	0.994392			0.005519
		E1-1–4	Who is the president now? (Xi Jinping)	0.994382			0.005528
		E1-1–5	What year is the lunar calendar this year?(Year of the Dog) [or: What is this year?]	0.991685			0.008183
		E1-7	How much do you have left if you have 100 yuan and spend 7 yuan?	0.988574			0.011245
		E1-8–1	Now I’ll say three words and listen carefully: apple/table/coin. Please repeat it	0.863059			0.134763
		E1-8–2	Please repeat the three words that I have just told you.	0.920389			0.078345
	Depression and Loneliness	E2-1	Did you find yourself in a good mood this past week?	0.948251		0.333655	0.050926
		E2-2	Have you felt lonely this past week?	0.978622			0.021038
		E2-3	Did you feel sad in the past week?	0.979504			0.020170
		E2-4	Do you think your life has been good this past week?	0.957045			0.042272
		E2-5	Did you feel like you didn’t want to eat in the past week?	0.980824			0.018871
		E2-6	Did you sleep badly in the past week?	0.976539			0.023088
		E2-7	Did you feel worthless this past week?	0.973585			0.025995
		E2-8	Did you feel like you had nothing to do in the past week?	0.976638			0.022990
		E2-9	In the past week do you think there are a lot of fun in life (interesting).	0.961065			0.038316
		E2-10	Did you feel lonely this past week?	0.971767			0.027784
		E2-11	Did you feel neglected by others this past week?	0.977821			0.021826
		E2-12	Have you felt isolated from others in the past week?	0.979291			0.020380
	Social adjustment	E7-1	If I have the opportunity, I would like to participate some work in the village / neighborhood committee.	0.981066		0.124563	0.018633
		E7-2	I often want to do something more for the society.	0.983329			0.016406
		E7-3	I like learning now.	0.985239			0.014526
		E7-4	I think I’m still a useful person to society.	0.983902			0.015842
		E7-5	Society is changing so fast that it is difficult for me to adapt to the change.	0.984406			0.015346
		E7-6	Society is changing so fast that it is difficult for me to adapt to the change.	0.984415			0.015337
		E7-7	More and more new social policies today are difficult for me to accept today.	0.986189			0.013592
		E7-8	Now social changes are increasingly detrimental for the elderly.	0.984878			0.014882
	Filial Piety Concept	E6-1	Bring up sons to support parents in their old age.	0.993233		0.047006	0.006659
		E6-2	Children should do something to make their parents proud.	0.994713			0.005203
		E6-3	Children should be grateful for their parents’ kindness.	0.995690			0.004241
		E6-4	Children should have the responsibility to support their parents.	0.995578			0.004352
		E6-5	When necessary, children should sacrifice for their parents.	0.989074			0.010752
		E6-6	In any case, the authority of the parents in the home should be respected by their children.	0.994793			0.005124
		E6-7	The most important thing for children to be filial to their parents is that their children are promising, Don’t let the elderly worry.	0.993529			0.006368
		E6-8	Children’s emotional care for their parents is more important than their financial support.	0.995625			0.004306

## Abbreviations

CLASS: China Longitudinal Aging Social Survey
CHDI: Cumulative Health Deficit Index
II: Personal income
EN: Empty nest
Aged 80+: Elderly aged 80 and over
FI: Household income
PLT: Person living together
INS: Proportion participating insurance
DP: Density of population
QA: Quality of air
NC: Proportion of only child
UR: Urbanization rate
